# Psychiatric morbidity of overseas patients in inner London: A hospital based study

**DOI:** 10.1186/1744-859X-4-4

**Published:** 2005-02-14

**Authors:** Fredy J Carranza, Alice M Parshall

**Affiliations:** 1 Adult Psychiatry, Central and North West London Mental Health NHS Trust, London, SW1V-2RH, UK; 2 Department of Adult Psychiatry, West London NHS Trust, Isleworth, TW7-6AF, UK

## Abstract

**Background:**

Evaluation of the referral, admission, treatment, and outcome of overseas patients admitted to a psychiatric hospital in central London. Ethical, legal and economic implications, and the involvement of consulates in the admission process, are discussed.

**Method:**

Assessment and review of overseas patients admitted between 1 January 1999 and 31 December 1999. Non-parametric statistical tests were used, and relevant outcomes described.

**Results:**

19% of admissions were overseas patients. Mean age was 38 years. 90% were unattached; 84% were white, 71% from European countries. 45% spoke fluent English. Differences in socio-economic status between home country and England were found. 74% were unwell on arrival; 65% travelled to England as tourists.

65% of admissions came via the police. 32% had been ill for more than one year before admission; 68% had psychiatric history. 77% were admitted and 48% discharged under section of the Mental Health Act. 74% had psychotic disorders, all of them with positive symptoms. 55% showed little to moderate improvement in mental state; 10% were on Enhanced Care Programme Approach. Relatives of 48% of patients were contacted.

The Hospital repatriated 52% of patients; the Mental Health Team followed up 13% of those discharged. The average length of admission was 43.4 days (range 1–365). Total cost of admissions was GBP350, 600 ($577, 490); average individual cost was GBP11, 116 (range GBP200-81, 000).

**Conclusions:**

Mentally ill overseas individuals are a vulnerable group that need recognition by health organisations to adapt current practice to better serve their needs. The involvement of consulates needs further evaluation.

## Background

Major cities in countries with religious, economical, or tourist attractions have experienced an increase in the influx of visitors; some of whom are mentally unwell, or subsequently become ill whilst in a foreign country.

Ødegaard (1932) described the tendency to travel in people with schizophrenia; more recent literature describe "crisis-flight" as a way of finding a geographical solution to internal problems [1], and airports as concrete representation of subjective conflicts related to separation and reunion at times of crisis [2]. Mental health care models of delivery, such as de-institutionalisation, the legal framework for admissions, the social acceptance of mental illness (including stigma and alienation) vary across countries world-wide [3]. Moreover, the perception and experience by vulnerable individuals of these issues in their own country might also be factors that contribute to individuals with mental illness travelling abroad.

Psychological [4], artistic -"Stendhal syndrome" [5], religious -"Jerusalem syndrome" [6], and time zone changes [7], among others, are described as factors related to psychiatric decompensation in travellers.

There is little data to show the number of these patients admitted to National Health Service (NHS) hospitals in the United Kingdom (UK), therefore it is difficult to know the real impact of overseas patients' admissions on the NHS.

This study describes the different aspects concerning the referral, admission, treatment, and outcome of overseas visitors (persons who are not ordinarily resident in the UK) admitted under a Mental Health Team (MHT) at a NHS psychiatric hospital in inner London. Ethical, legal, and economic implications are discussed. The involvement of consulates in the admission process of overseas patients is suggested and the benefits of their involvement discussed.

### Setting

The multidisciplinary MHT for this study serves a population of around 29, 000 local residents, in addition to the homeless and transient people in the area of Westminster in central London. The area is close to major international rail and bus terminals, has direct connection with large international airports, and has a number of business and tourist attractions. There are mixed affluent and under-privileged sectors in the area with an average of 41 psychiatric beds for 100, 000 habitants, and a Jarman index (an index of social deprivation, ranging from -32.79 [less deprivation] to 54.89 [more deprivation]) of 22.7. The MHT had a number of beds allocated for admission at the 75-bed psychiatric hospital (the Hospital), which is also used by other mental health teams operating in annexed geographical areas.

## Methods

Review of all overseas nationals between 18–65 years of age, admitted to Hospital between 1 January 1999 and 31 December 1999. The sample included patients admitted before 1 January 1999 who were still inpatients by 31 December 1999. Foreign residents in the UK, transient foreign nationals attending outpatient clinics, foreign nationals pursuing immigration into the UK, or patients seeking, or under refugee status were not included in this study.

The medical team assigned diagnoses using the ICD-10 (Classification of mental and behavioural disorders: clinical description and diagnostic guidelines. WHO, Geneva, 1992), and additionally using the ICD-10: DCR-10 (Classification of mental and behavioural disorders: diagnostic criteria for research. WHO, Geneva, 1993). Both authors, FJC and AMP, were directly involved in the management of the patients in this study.

Data was obtained from:

• Medical notes.

• Discharge summaries from previous admission in the UK (if applicable).

• Medical and psychiatric reports from patients' country of origin (if applicable).

• Database archives of the Mental Health Team.

• The Hospital's Human Resources department.

• Social Services reports.

• Police reports.

• Assessment and interview of patients and relatives (when available), by AMP and FJC.

Fisher's exact test was used in the statistical analysis to examine the relationship between two categorical variables. The relationship between cost and other continuous variables was measured using Spearman's rank correlation test. The relationship between cost and categorical variables was assessed using the Mann-Whitney U test.

## Results

### Demographic characteristics (Table 1)

**Table 1:** Demographic characteristics

		**No of patients (n = 31)**	**%**
**Gender**			
Female		13	42
Male		18	58

**Age (years)**			
Range	23–52		
Mean	38		
Mean male age	35		
Mean female age	41		

**Marital status**			
Single	(14 male-7 female)	21	68
Married	(all male)	3	10
Divorced	(all female)	6	19
Widowed	(male)	1	3

**Nationality**			
EU nationals (includes five with adopted EU nationality)		22	71
Other nationalities		9	29

**Ethnicity**			
White		26	84
Black		3	10
Other		2	6

**Language**			
Did not speak English		2	6
Spoke basic English		15	48
Spoke fluent English		14	45
			
Required interpreter		17	55
Did not require interpreter		14	45

**Mobility before arrival in England**			
Travelled directly to England		21	68
Travelled to other countries before arriving in England		10	32

**Mental health on arrival in England**			
Unwell on arrival		23	74
Became unwell in England		4	13
Not ascertained		4	13

**Purpose of travel to England**			
Tourism		20	65
To "escape persecution" in their country		5	16
To visit friends-relatives		5	16
Business		1	3

**Support in England (other than statutory services)**			
None		26	84
From friends or relatives		5	16

Of 163 (100%) admissions under the care of the MHT between 1 January 1999 and 31 December 1999, 31 (19%) were overseas patients. 58% were male; age range (years) was 23–52. 90% were unattached. 71% came from Europe; most were white (84%). 45% spoke fluent English, 48% spoke basic English; 55% required an interpreter for assessments.

68% travelled directly from their home country to England; 32% had been to other countries before arriving in England. 74% were mentally unwell on arrival in England. 65% travelled as tourists; 16% gave "escaping persecution" as a reason for travelling. Only 16% had support from friends or relatives in England.

The socio-economic status of overseas patients in their home country showed one (3%) homeless and 97% housed. Of these, 13 patients lived independently, 13 lived with relatives, and 4 were housed by social services. 52% had been employed and 48% unemployed, with 4 of them receiving social benefits.

In England, 61% overseas patients were homeless, 13% were housed by local services, and 26% lived in rented accommodation, with relatives, or with friends. 10% had financial income from employment, 3% received benefits, 26% received financial help from family or other sources, and 61% patients had no financial income.

### Admission, assessment and treatment (Table 2)

**Table 2:** Admission, assessment and treatment

		**No of patients (n = 31)**	**%**
**Mode of contact with the Mental Health Team**			
Police referral to mental health team for assessment		13	42
Police referral to hospital (section 136 of the Mental Health Act)		7	23
Assessment by mental health team (community-hospital)		11	35

**Appeals against section of the Mental Health Act**			
Appealed		9	29
Tribunals (5 patients)	7		
Not discharged	6		
Deferred discharge	1		
Discharged from section by MHT before hearing	3		

**Symptoms on admission**			
Delusions-hallucinations-thought disorder		25	81
Mania-hypomania-elated mood		4	13
Depression-delusions		2	6
Impaired insight		25	81

**Length of illness before admission**			
>1 year		10	32
6–12 months		7	23
1–5 months		8	26
Not ascertained		6	19

**Psychiatric history**			
Had contact with psychiatrist		21	68
>1 year before admission (range 1–10 years)	14		
3 months before admission	7		
None		4	13
Known to social-primary care, but not to psychiatric team		2	6
Not ascertained		4	13

**Dual diagnosis**			
Diagnosed		0	00
History of drug use		3	10
Used drugs regularly		1	3

**Forensic history**			
Had history		6	19
No history		17	55
Not ascertained		8	26

**Medication**			
Refused, or given "if required"		5	16
Given regularly		26	84
Atypical neuroleptics	15		
Typical neuroleptics	10		
Antidepressants	1		
Took medication in the past		15	48

Forty two per cent of patients were referred to the MHT for assessment at a police station. The police brought 23% of patients to Hospital, for assessment under section of the Mental Health Act 1983 (MHA) – see Table 4 for further explanation of relevant sections of the MHA. 35% were assessed in the community or self-presented to hospital.

No immediate discharges were granted on seven appeal hearings to review formal admissions; one (3%) patient received a deferred discharge. 81% presented with delusions, hallucinations, or thought disorder, alone or in combination. 81% had impaired insight. 32% had been ill for at least one year before the current admission. 68% had a psychiatric history, 13% had no psychiatric history; 6% were known to social and primary care services, but had not been assessed by a psychiatric team.

Table 3 shows the diagnoses according to the International Classification of Diseases-10^th ^edition (WHO, Geneva 1992). 74% had psychotic disorders, all of them with positive symptoms of the illness.

**Table 3:** Diagnosis

**Diagnosis**	**ICD-10 (WHO) classification**	**Number of patients**	**Total (%)**
Schizophrenia	F 20.0	16	
	F 20.00	1	
	F 20.02	1	18 (58)
Acute psychotic disorder	F 23.2	2	
	F 23.9	2	4 (13)
Schizoaffective disorder	F 25.2	1	1 (3)
Bipolar affective disorder	F 30.1	1	
	F 31.2	5	6 (19)
Drug induced psychosis	F 14.55	1	1 (3)
Not determined	-	1	1 (3)

**Table 4:** Discharge and outcome

		**No of patients (n = 31)**	**%**
**Mental state on discharge**			
No or little improvement		6	19
Moderate improvement		11	35
Major improvement		14	45

**Contact with relatives-care team in country of origin**			
Before admission		1	3
At some point after admission		23	74
With care team	18 patients		
With family	14 patients		
With care team and family	9 patients		
No contact made		7	23

**Contact with consulates-embassies**			
Contacted		16	52
Gave information	9		
Provided travel documents	4		
Could not help	3		
Not contacted		15	48

**Care Programme Approach**			
Enhanced CPA		3	10
Follow up by mental health team	2		
Initiated but discontinued	1		
Standard CPA		28	90

**Patients-relatives agreement with discharge plan**			
Agreed		27	87
Disagreed		4	13
Absent without leave	2		
Deferred discharge by MHRT	1		
Ongoing review under s.86 MHA*	1		

**Outcome on discharge**			
Repatriated by the hospital		16	52
Discharged to return to country of origin		6	19
Taken home by relatives		2	6
Absent without leave		2	6
Discharged with follow up by the Mental Health Team		4	13
Application made for section 86 MHA*		1	3

**Medication on discharge**			
Supplied to take home		25	81
Not supplied		6	19
Unreliable	4		
absent without leave	2		

**Average length of treatment (days)**	43.4		
	Range 1–365		

**Length of treatment according to Mental Health Act status**			
Voluntary (mean 22.3 days)		7	23
Section 4** (mean 4 days)		1	3
Section 2*** (mean 21 days)		13	42
Section 3**** (mean 91.5 days)		10	32

*Section 86 of the Mental Health Act 1983: Allows the Home Secretary to authorise the removal to another country of patients, who are neither British nor Commonwealth citizens having the right of abode in the UK, who are receiving treatment for mental illness in hospital under section of the MHA.

**Section 4: compulsory admission and detention for up to 72 hours for assessment.

***Section 2: compulsory admission and detention for up to 28 days for assessment or assessment followed by treatment for mental disorder.

****Section 3: compulsory detention for up to six months for treatment.

One (3%) patient used drugs regularly, 10% had a history of drug use. There was no dual diagnosis. 55% had no forensic history; one patient was referred to the MHT by the local forensic team. On admission, 84% took medication regularly; 16% refused or had medication "If required", usually for agitation. 48% had taken medication for mental health problems in the past.

Two patients had been admitted under the MHT on a previous visit to London; at that time they had been repatriated and subsequently admitted to hospital in their country, returning back to London after discharge from hospital. One patient had been admitted to two other psychiatric hospitals in London before admission to the MHT. One patient had been assessed by the MHT on a previous visit to London.

**Figure 1:**
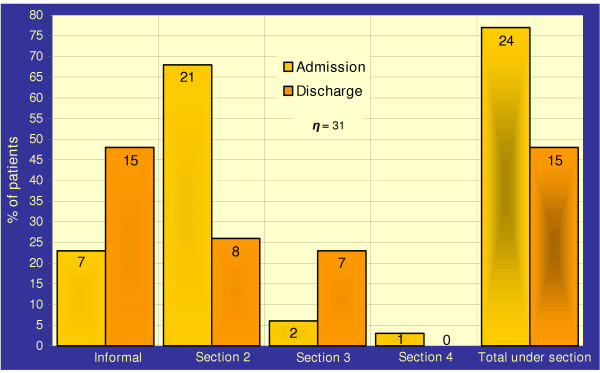
Mental Health Act 1983 status on admission and discharge

Figure 1 shows the MHA status on admission and discharge, and the sections of the MHA used. 77% of patients, including two patients admitted informally and placed under section of the MHA shortly after admission, were admitted and 48% were discharged under section of the MHA.

### Discharge and outcome (Table 4)

Nineteen per cent of patients showed no-little improvement in mental state; 35% showed moderate improvement, 45% showed a major improvement. The mental state was assessed regularly at weekly review meetings. No outcome scales were used. The presence of insight was taken as indicator of major improvement.

One (3%) patient's relatives were contacted before admission; relatives of 45% of patients were contacted at some point after admission. Consulates of 52% of patients were contacted, most of them provided information, and in some cases supplied emergency travel documents.

10% were on Enhanced and 90% on Standard Care Programme Approach (CPA), the statutory planning and provision of mental health and social after-care. The MHT followed up 10% of patients after discharge (two on Enhanced and one on Standard CPA). Agreement with patients and/or relatives to a discharge plan was achieved in 87% of cases.

52% of patients were repatriated by the Hospital. These took place by air, accompanied by two members of staff, following the Hospital policy. 19% made their own arrangements to return home after discharge; relatives took 6% home. The MHT organised follow up for four patients, of these one decided to return home after the persecutory delusions had subsided. 81% of patients were supplied with medication (usually a two weeks supply) to take home.

The average length of treatment in Hospital was 43.4 days per individual (range 1–365 days). One patient had been admitted before 1.1.1999 and was still admitted by the 31.12.1999. Patients under section 3 of the MHA spent the longest in Hospital (mean 91.5 days). Voluntary patients and those under section 2 spent similar numbers of days in Hospital (mean 22.3 and 21 days respectively).

The total cost of the 31 admissions of overseas patients was GBP350, 600 ($577, 490). The average individual cost of admission was GBP11, 116 ($18, 230); the range was GBP200 – GBP81, 000. The costs were for nursing care and repatriation. Other costs, such as translators, special nursing observations, or legal costs, were not included.

Spearman's rank correlation test showed a highly significant positive correlation between length of admission and cost (P < 0.01). Mann-Whitney U tests showed a significant difference in cost between patients with and without housing in England (P = 0.02), and between patients with and without financial help in England (P = 0.01). Patients with housing had a median cost of GBP4, 500 compared to GBP11, 000 for those without housing; patients with financial help had a median cost of GBP4, 500 compared to GBP12, 000 for those without help.

## Discussion

Overseas patients form a significant proportion (19%) of the admissions under the care of the MHT. It is estimated that overseas patients account for 10% of admissions in central London [8], whilst research in the same geographical area as this study report rates of 16% [9]. Studies in Jerusalem, where all psychiatric admissions of tourists are channelled into one central hospital, report an average of 40–50 admissions a year [4-10], whilst in Florence 107 tourists were admitted to a central hospital between 1978 and 1986 [5].

Homelessness in England among overseas patients in this study (61%) differs significantly from rates of homelessness among local (3%) (Parshall & Carranza, European Congress of Psychiatry, Madrid, 2001), and other patients admitted in Westminster -25% [11].

Geographical mobility has been linked to disruption in the continuity of care of patients, lack of accountability in census figures [12-14], and for service planning and provision [15]. These problems also apply to overseas patients, whose mobility is likely to have influenced the length of untreated illness and the level of contact with health services before admission. This may be illustrated by four overseas patients' previous contacts with mental health services in London, which resembles the "revolving door" phenomenon, widespread in psychiatric services in England.

A comparison of UK and European studies on attitudes towards the mentally ill describes British respondents as one of the most tolerant with little fear of the mentally ill, who consider mental illness as a universal condition, and favour community-based interventions as opposed to institutionalised care [16]. The perception of British attitudes towards mental illness, coupled with some familiarity with the English language, may have encouraged an "international drift" to the United Kingdom in individuals already unwell. In this study no specific factors could be identified as causes for overseas patients' mental breakdown.

Police involvement in the referral process is a significant predictor of admission to psychiatric hospitals [17]. Overseas patients assessments under section 136 of the MHA (Table 2) are likely to contribute significantly to the rate of these referrals to the psychiatric services in Westminster reported as one of the highest in the United Kingdom [18]. Fisher's exact test showed a significant association between mode of contact with the MHT and MHA status on admission (P < 0.001), with only 5% voluntary hospitalisations via the police, compared to 73% voluntary admissions via the MHT. The proportion of overseas patients' admissions via the police (65%) (Table 2) is similar to reports from London [19], and Jerusalem [4], and differs from rates reported among UK (24%) and local patients (6%) admitted under the MHT (Parshall & Carranza, European Congress of Psychiatry, Madrid, 2001). Offences by overseas patients leading to contact with the police were mainly behavioural and non-violent (e.g. bizarre conduct in public places, or not paying fees for services). One overseas patient was admitted via the Court Liaison Service, compared to the reported 15% of other admissions to the MHT from that service [20].

Overseas patients' admissions under section of the MHA (77%) correspond with reports of admissions from Heathrow airport -81% [7], 69% [19], and the local Hospital – 76% (Hospital MHA Officer's data), and differ from rates for England, where less than one third of admissions are under the MHA [21].

Rates of overseas patients with schizophrenia or related disorders (74%) (Table 3) are comparable to figures from studies of travellers in New York -74% [2], London -50% [7], 46% [19], Jerusalem -63% [10], 85% [4], and Florence -68% [5]. These rates differ from figures of admissions with schizophrenic psychosis in inner London -30% [22], and Westminster -38% [23].

All overseas patients with schizophrenia presented with "positive symptoms" (delusions, hallucinations, and thought disorder). These are prevalent in urban populations with schizophrenia [12], and have been associated with high mobility [24] and homelessness [25]. "Negative symptoms" such as marked apathy, paucity of speech, blunting or incongruity of emotional responses (ICD-10: DCR-10) are associated with prefrontal dysfunction [26], and impairment of brain executive functions [27]. Patients with negative symptoms may find the planning and execution of foreign trips too challenging, and might also explain their absence in this study.

Monopolar depression, personality disorder, neurotic or stress related disorders, or disorders other than the ones shown in Table 3, were not found in this study. The absence of patients with dual diagnosis (substance misuse problems and mental illness in the same patient at the same time) contrasts with reports of 50% substance use among the mentally ill in the UK and substance misuse problems in 36% of patients with psychosis in London [28].

The low number of patients on the Enhanced component of the CPA, reflects the difficulties found on establishing responsibilities for the provision of services and care planning in overseas patients, and misrepresents the severity of the problems with which these patients present. A limiting factor is the difficulty in setting up care plans for patients whose aftercare is to be implemented by agencies abroad.

Mental Health Review Tribunals and Managers' Hearings discharged no overseas patients. Figures for England and Wales show discharge rates between 14.4% and 15.6% [21] and 7.0% in high security hospitals [29]. Discharge from hospital on grounds other than medical (e.g. request for repatriation by relatives) may explain the percentage of overseas patients discharged with little or moderate improvement in mental state (55%), and discharges from hospital under section of the MHA (48%) (Figure 1).

Overseas patients' refusal to return to their country, where a health and care system may or may not be in place, poses an ethical and legal challenge to services. Section 86 of the MHA (see Table 4) is rarely used, perhaps due to the lengthy process and the varied factors to consider for its application. The Department of Health's recommendation to treat patients as close to home as possible [30], and the need for a "substrate for health" -looking not only at psychiatric interventions, but also at the individual's basic needs, housing, and a social network [31], need careful consideration when making decisions on repatriation.

Since October 2000 contravention against the European Convention on Human Rights (ECHR) [32] can be challenged in UK courts. Problems with language translation and interpretation, usually evident on admission coincidental with an acute stage of patients' mental state, are common when treating overseas patients. These can give rise to ethical and legal issues for example, when assessing capacity and consent to treatment. Current legislation states that all patients should be given information both orally and in writing on their legal position and rights (MHA)[33], of the reasons for their detention (ECHR [32], Mental Health Act Code of Practice [34]) but section 132 of the MHA is silent on this point, in a language that the person understands (ECHR)[32]. Failure to do so may be challenged under article 5(2) of the ECHR.

Particularly relevant to overseas patients is the issue of deportation under section 86 of the MHA, which may be challenged under article 3 of the ECHR. Delays on discharging a patient because of failure to set-up aftercare services may breach article 5(4). Difficulty of access to information on the grounds for detention to apply for a hearing may breach article 6. Discrimination in the provision of services, such as individual therapies, multidisciplinary team involvement, or treatment in locked units may breach article 14 of the Act.

The Eighth Principle of the Data Protection Act 1998 -personal data should not be transferred outside the European Economic Area unless that country ensures its adequate protection [35], is difficult to guarantee when dealing with foreign agencies on behalf of patients, and may give rise to breach of article 8(2) of the ECHR. Conversely, the lack of consultation and provision of information to a nearest relative on patients' admissions may be challenged under the same article 8(2).

The Council of Europe determines that family and other people close to a patient should be consulted on involuntary placement and treatment [36]. The MHA provides legislation on ascertaining the nearest relative of patients from England and Wales, but gives no indication on how to proceed in the case of foreign nationals. The lack of nearest relative in overseas patients has ethical and legal implications, particularly on issues of risk assessment, information about their power to discharge a patient, to delegate their role, advanced directives, and repatriation.

At present, consular representations play, to a major or lesser degree and at an informal level, a role in some ways similar to that of a nearest relative, which is not recognised by mental health law. Contact with embassies is described as ranging from lack of involvement, particularly when patients are in need of repatriation [8], to full cooperation with contact and liaison with services abroad, particularly from European embassies [37]. A way forward for future legislation could be for the consular representations to take formally the role of nearest relative, which could revert back to the patient's relatives when practicable. The Expert Committee Review of the MHA recommends that the powers of the nearest relative should be reduced and for the provision of advocates independent from the service provider [38]. Proposals in the Government's Draft Mental Health Bill include the patient's choice of a "nominated person" to replace the figure of nearest relative, and a duty to provide sufficient advocates [39]. A feasible option would be for consulates to fulfil the role of nearest relative, which would automatically encompass the role of advocate; the advantages include:

• The prompt nomination of a nearest relative when it is not possible to identify one, or when they have been displaced of their role by the Court.

• To prevent problems with confidentiality e.g. when trying to contact relatives, who may not speak English, and services abroad.

• Provisions under the MHA do not apply to voluntary patients; thus they may receive less information on issues related to their admission. In these patients, as in detained patients, consulates could be useful in establishing links locally, with services abroad, and as a reference point e.g. in overseas patients missing in their country who present to health services abroad.

• Admissions under the MHA require the involvement of social services. There may be a negative perception or reluctance to accept the input from social services by patients when the Court appoints a social worker as the nearest relative, e.g. when a relative cannot be identified.

• As advocates, consulates are better prepared to assist patients with lessening the impact of transcultural barriers, relaying information, which could assist patients on making decisions e.g. on medico-legal matters.

• From the patient's perspective, familiarity with the person representing the nearest relative may reassure them on issues of the service's independence and lack of bias, leading to better co-operation with their treatment and care plans.

The pressure on mental health services in inner London may be a consequence of changes in patients' characteristics- younger, increasingly mobile, more likely to be unattached and unemployed [40], features that also correspond with the average patient's profile in this study (Table 1). Furthermore, patients with these characteristics who are less able to live independently increase the costs of care [41]. Likewise, overseas patients have a high degree of dependence on care services, and their high mobility is likely to have an influence on levels of provision and possibly on the reported underestimate of needs in inner London by measures of service requirement, such as the Mental Illness Needs Index (MINI) [42]. Mobility is also likely to be an obstacle for overseas patients' inclusion in audit, service planning, and mental health strategies aimed at improving standards of care.

## Conclusions

The sample size in this study is small, which makes our findings difficult to generalise. The figures in this paper represent the results of one mental health team, among the more than 50 mental health teams in central London, which suggests a higher scale to this problem. Research is much needed in this area.

Our findings replicate at international level the "social drift" seen in people affected by psychiatric morbidity into deprived inner city areas [43]. A high proportion of patients in this study, particularly patients with schizophrenia, fall into what has been described as "double drift" [44], by virtue of moving from one country to another, and then into a socially isolated urban area where they become part of a low socio-economic group.

High mobility among overseas patients had a marked impact on homelessness, contact with services, care and service planning and delivery, Mental Health Act reviews' outcomes and status on admission and discharge. Psychotic disorders with positive symptoms were prevalent. Police involvement in the referral process was high, correlated positively with the high rate of involuntary admissions, and negatively with the type of offences attributed to these patients. A highly significant correlation was observed between length of admission and cost, with a significant cost difference between overseas patients with and without social and financial support.

An enhanced role for consulates as representative bodies for overseas patients receiving psychiatric treatment needs to be explored and formalised.

Service providers need mechanisms better able to identify and to evaluate overseas patients' needs. This would allow patients' data to count in audit, research, and financial planning; thus facilitating their inclusion in user and information groups, and strategies aimed at improving standards of care.

Recent changes to the Charging Regulations for treatment under the NHS of non-resident patients [45] need to take into account the characteristics and problems common to overseas patients with psychiatric illnesses and to adapt legislation accordingly.

As the boundaries between domestic and international health matters become blurred, countries need to pursue a global integration of policies aimed at helping people with mental illness in general, and patients with high mobility in particular.

## Competing interests

The author(s) declare that they have no competing interests.

## Authors' contributions

FJC conceived the study, collected data and drafted the manuscript. AMP participated in the design of the study and reviewed the manuscript. Both authors read and approved the final manuscript.
